# A Novel Distribution for Representation of 6D Pose Uncertainty

**DOI:** 10.3390/mi13010126

**Published:** 2022-01-13

**Authors:** Lei Zhang, Huiliang Shang, Yandan Lin

**Affiliations:** 1 Academy for Engineering and Technology, Fudan University, Shanghai 200433, China; leizhang18@fudan.edu.cn; 2 School of Information Science and Technology, Fudan University, Shanghai 200433, China; shanghl@fudan.edu.cn; 3 Institute for Electric Light Sources, School of Information Science and Technology, Fudan University, Shanghai 200433, China

**Keywords:** probability theory, dual quaternion, pose uncertainty, lie group, Bingham distribution

## Abstract

The 6D Pose estimation is a crux in many applications, such as visual perception, autonomous navigation, and spacecraft motion. For robotic grasping, the cluttered and self-occlusion scenarios bring new challenges to the this field. Currently, society uses CNNs to solve this problem. The CNN models will suffer high uncertainty caused by the environmental factors and the object itself. These models usually maintain a Gaussian distribution, which is not suitable for the underlying manifold structure of the pose. Many works decouple rotation from the translation and quantify rotational uncertainty. Only a few works pay attention to the uncertainty of the 6D pose. This work proposes a distribution that can capture the uncertainty of the 6D pose parameterized by the dual quaternions, meanwhile, the proposed distribution takes the periodic nature of the underlying structure into account. The presented results include the normalization constant computation and parameter estimation techniques of the distribution. This work shows the benefits of the proposed distribution, which provides a more realistic explanation for the uncertainty in the 6D pose and eliminates the drawback inherited from the planar rigid motion.

## 1. Introduction

The Pose Estimation of rigid objects has a wide range of applications in today’s life, such as robot grasping [1], aerospace applications, autonomous driving [2], and so on.

**Figure 1:**
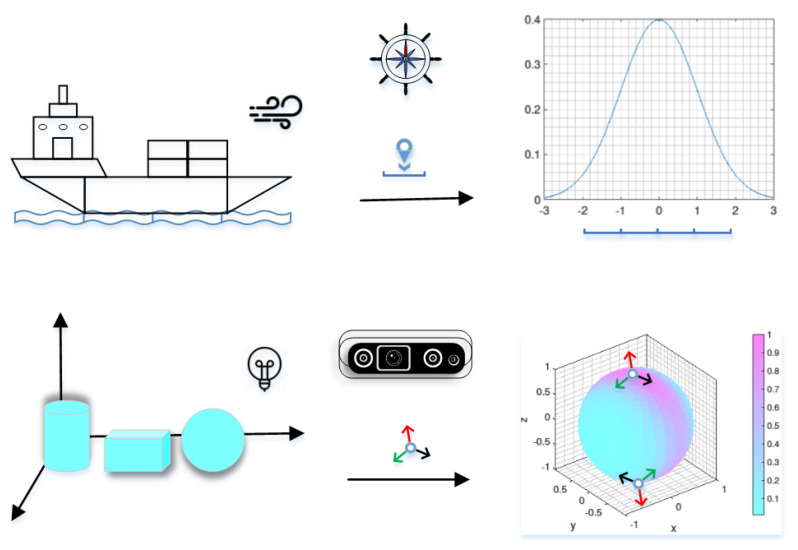
The analogy to localize a ship moves on the sea. The position appears as the Gaussian distribution with noise measurements caused by the wind and waves. However, it is no longer suitable for the 6D pose uncertainty influenced by illumination and symmetric objects themselves. The result of the proposed distribution shows an antipodal symmetry for the 6D pose.

Throughout history, pose estimation has played a vital role, from Ceres’s position predicting to navigation on the sea ([3], Introduction 1.1). One day, *Captain cook* sailing in the sea has lost his bearings. The ship moves on a wavy and wind sea. With the reasonable assumption, He estimates the optimal localization of the ship from the noisy measurements (Figure 1, top).

New challenges come to this field. The success of service robotics relies on well-perceiving an unfamiliar kitchen scenarios, such as bottles, dishes, and containers. Robot grasps under cluttered or limited lighting environments are becoming common in a rapidly growing warehouse automation industry [4]. Consequently, robots need to know the full 6D pose of objects to manipulate objects, which is difficult due to the uncertainty caused by environmental factors and sensing technologies. The intuition is to deploy expensive high-precision sensors to avoid such uncertainty. However, all sensors have limited precision, and measurements derived from actual sensors have associated uncertainty ([3], Introduction 1.2).

The CNN [5] broke through in the ImageNet [6] challenge, motivating more and more works to embrace deep learning. However, most prior works in this area failed to take the weak information [7] and the 6D pose uncertainty into account, and they only provide a single best guess for each object pose [8,9,10]. The accurate metric of the model cannot handle unprepared situations, such as dim lighting. The uncertainty will rise with the increase in accuracy over time.

The pose estimation of symmetric objects is currently the most challenging task [11]. Existing multiple correct poses for the same visual appearance [12]. This issue also results in lousy training performance since a network receives inconsistent loss signals. Xiang et al. [9] design an MSE loss function based on quaternions and regress the 3D rotation using the neural network. It reports better performance for symmetric objects such as bowls. However, they do not take the underlying structure of the manifolds into consideration.

Deng et al. [13] detect the object’s position by a bounding box using a neural network. They assume the Gaussian position and approximate the observation likelihood to obtain the conditioning orientation density by a Rao–Blackwellized particle filter. The result is considerably robust in rotational uncertainty. However, this approach is computationally expensive, and it is not clear which kind of distribution holds on the orientations. Furthermore, uncertain rotation axes need to be considered when underlying uncertainty is not axis aligned [14].

Nonetheless, the neural network correctly assumes the underlying structure of the data is robust. Prokudin [15] exhibits a good example dealing with periodic circular data. The same loss will emerge when the predicted angle is equal to 1° or 359° with the ground truth angle of 0°. He presents a loss based on the directional setting and assumes the angle obeys the von Mises distribution. As a result, the model works suitably for the data with the periodic nature.

The Gaussian assumption is commonly embedded, leading the neural network to fail to learn the underlying manifold structure of the 6D pose. Holding the robust assumption of uncertainty is the foremost for 6D pose estimation.

Inevitably, coupling or decoupling is the first consideration when parametrizing the 6D pose. It is also related to further modeling uncertainty. Daniilidis [16] employs dual quaternion to parameterize the 6D pose for hand–eye calibration and establishes a linear homogeneous system, simultaneously solving rotation and translation. Goddard and Addi [17] capture the correlation between rotation and translation for rigid motion tracking by dual quaternions.

Horn [18] proposes the method that decouples the orientation from translation. Inspired by Horn, Srivatsan [19] makes a linear assumption on the state of the SE(3) elements by adopting dual quaternions. Li [20] explains the correlation between rotation and translation terms based on hyperspherical parallel transport and gives a Bingham distribution on the orientation decoupled from the translation. Manhardt [12] illustrates the rotation uncertainty caused by occlusion in the 6D pose estimation by employing Bingham distribution. However, it is still unknown how the Bingham distribution extends to SE(3) space without variations.

The work’s most Bingham distribution of interest is motivated by Glover. He presents the BPA [21] algorithm to recover the full poses from patches of local features of the 3D point cloud. This work extends from the SE(2) [22].

### Main Contribution

In this work, we aim to model the uncertain 6D pose by extending the probability density function on the SE(2) to the SE(3). This is achieved through parameterizing the 6D pose by dual quaternions. The work simplifies a future generalization to the 6D pose estimation of objects (Figure 1, bottom). The proposed distribution appears as a marginal distribution for the rotational part of the proposed model. We further discuss the relationship between orientation and position. To the best of the author’s knowledge, there is no work exactly claimed for this.

This paper is organized as follows. We will compare several existing 6D parameterization techniques and select the most promising one in Section 2. In Section 3, we propose a new probability distribution on the 6D pose. The normalization constant and parameter estimation techniques will be discussed. Later, in Section 4, we use the proposed framework to represent the transformation of the 6D pose, and the algorithm that recovers the transformation from the samples of our proposed distribution will be presented. Lastly, the discussion and conclusions of this work are in Section 5.

## 2. Preliminaries

There are several existing methods to parameterize SE(3) elements, including orthonormal rotation matrices, Euler angles, Rodrigues vectors, quaternions, etc. For orthonormal rotation matrices, the compounding of two elements is much more complex ([23], p. 45). Although Euler angles are invariant under transforms and easy to comprehend, there exist singularities [24]. Rodrigues vectors are complex to implement as a composition algorithm [25]. For parameterization of the 3D rotation, the quaternion is the best from the analog computation view [26]. Still, it is also limited to representing rotation in a full 6D pose, and the translation must be dealt with separately.

The dual quaternions this work introduced takes both rotation and translation into consideration. It provides a closed-form solution for the composition of 6D poses, which is the analogy to the transform matrix in homogeneous coordinates [27]. Kenwright [28] finds that the transforms by the dual quaternion multiplication 10 percent faster compared to matrix multiplication on average. Nonetheless, Kavan et al. [29] present a practical example, and they utilize dual quaternions to solve the shortage of the linear blend skinning.

**Conventions**. In this work, lower-case letters represent scalars, and matrices are represented by capital letters, vectors, and quaternions in bold. Quaternions are distinguished from dual quaternions by a caret, e.g., $\tilde{q}$ denotes a quaternion and $\hat{q}$ denotes a dual quaternion. Two quaternions $\tilde{p}$ and $\tilde{q}$ multiplication is denoted as $\tilde{p}⊙\tilde{q}$, while the multiplication of the two dual quaternions is denoted as $\hat{p}⊗\hat{q}$. The dot product and cross product of vectors v and w are denoted as 〈v,w〉 and v×w, respectively. Finally, we use H represent the skew-field of quaternions. With this in mind, consider the quaternions $\tilde{q}\in H$. The pose is synonymous with the 6D pose or transformation; the rotation is synonymous with orientation; the translation is synonymous with the position.

### 2.1. Quaternions

Similar to complex numbers, the sum of a real number and three complex numbers represent quaternions. The quaternion $\tilde{q}\in R^{4}$ can be treated as a 4-tuple $\left(q_{0},q_{1},q_{2},q_{3}\right)$. It can also be identified with the typical basis elements *i*, *j*, *k* via the coefficients:

(1) $$H=\{\tilde{q}\;|\;\tilde{q}=q_{0}+q_{1}i+q_{2}j+q_{3}k,\;q_{0},q_{1},q_{2},q_{3}\in R\}.$$

The multiplication of the two quaternions $\tilde{p}=\left(p_{0},p\right)$, $\tilde{q}=\left(q_{0},q\right)$ is given by:

(2) $$\tilde{p}⊙\tilde{q}=p_{0}q_{0}-\langle p,q\rangle +q_{0}p+p_{0}q+p\times q,$$

where $p_{0},q_{0}$ names the scalar part and $p,q\in R^{3}$ is the vector part; note that the product is not commutative.

Furthermore, linear operators [30] $Q_{p}^{+}\;\;\mathrm{and}\;\;Q_{q}^{-}$$\to R^{4\times 4}$ and associated with (2) can be also defined by matrix-vector form as:

(3) $$\tilde{p}⊙\tilde{q}=Q_{p}^{+}\cdot \tilde{q}=Q_{q}^{-}\cdot \tilde{p},$$

with

$$\begin{array}{c} Q_{p}^{+}=[\begin{array}{cc} p_{0} & -p^{T}\\ p & p^{\times }+p_{0}I_{3} \end{array}],Q_{q}^{-}=[\begin{array}{cc} q_{0} & -q^{T}\\ q & -q^{\times }+q_{0}I_{3} \end{array}]. \end{array}$$

where ${\left[\;\right]}^{\times }$ is the skew-symmetric matrix generated from the corresponding vector.

The canonical norm on H of quaternions is defined by $\left|\right|\tilde{q}\left|\right|=\sqrt{\tilde{q}⊙{\tilde{q}}^{*}}=\sqrt{{\tilde{q}}^{*}⊙\tilde{q}}=\sqrt{{q_{0}}^{2}+{q_{1}}^{2}+{q_{2}}^{2}+{q_{3}}^{2}}.$

The conjugate of the quaternion is obtained by changing the sign of each element in the imaginary part: ${\tilde{q}}^{*}=\left(q_{0},-q\right)=\left(q_{0},-q_{1},-q_{2},-q_{3}\right)$.

Last but not the least, the quaternion addition is simply the 4-tuple addition of quaternion representations: $\tilde{p}+\tilde{q}=\left(p_{0},p\right)+\left(q_{0},q\right)=\left(p_{0}+q_{0},p+q\right)$.

### 2.2. Representation of 3D Rotation

Unit quaternions $\tilde{q}$ are quaternions with $\left|\right|\tilde{q}\left|\right|=1$ and commonly represent the rotations in 3D Euclid space. The inverse of a unit quaternion is obtained by its conjugate form ${\tilde{q}}^{-1}={\tilde{q}}^{*}$.

The quaternion can represent the rotation around a 3D axis with a unit-length vector v and the rotation angle θ∈[−π,π]:

(4) $$\tilde{q}=cos\left(\frac{\theta }{2}\right)+vsin\left(\frac{\theta }{2}\right).$$

A point $p=\left(p_{1},p_{2},p_{3}\right)\in R^{3}$ is denoted as the purely imaginary quaternion without real part $\tilde{p}=p_{1}i+p_{2}j+p_{3}k$. From (4), the rotated point ${\tilde{p}}_{rot}$ is obtained as [25]:

$$\begin{array}{c} {\tilde{p}}_{rot}=\tilde{q}⊙\tilde{p}⊙{\tilde{q}}^{*}. \end{array}$$

In addition, $\tilde{q}$ and $-\tilde{q}$ represent the same rotation due to the *antipodal* property on the hypersphere $S^{3}$, so the set U of unit quaternion is a double coverage of the SO(3) of the 3D rotations.

### 2.3. Dual Quaternions

The dual theory is helpful when understanding the concept of dual quaternions. A dual number combines the non-dual part $a_{1}$, and the dual part $b_{1}$ is represented as $a_{1}+\epsilon b_{1},$ where $a_{1},b_{1}\in R$. The ϵ is the dual unit; note that ϵ≠0, $\epsilon^{2}=0$. The multiplication of two dual numbers is given as $\left(a_{1}+\epsilon b_{1}\right)\left(a_{2}+\epsilon b_{2}\right)=a_{1}a_{2}+\epsilon \left(a_{1}b_{2}+a_{2}b_{1}\right).$

Dual quaternions ($\hat{x}\in H_{D}$) are quaternions equivalent to dual numbers, i.e., replacing real numbers $a_{1},b_{1}$ with quaternions $\tilde{p}$ and $\tilde{q}$. Thus, a dual quaternion is given as follows:

(5) $$H_{D}=\left\{\hat{x}\;|\;\hat{x}=\tilde{p}+\epsilon \tilde{q},\;\tilde{p},\tilde{q}\in H\right\}.$$

The multiply operation of two dual quaternions is similar to dual numbers. As two dual quaternions ${\hat{x}}_{1}={\tilde{p}}_{1}+\epsilon {\tilde{q}}_{1}$ and ${\hat{x}}_{2}={\tilde{p}}_{2}+\epsilon {\tilde{q}}_{2}$ (${\hat{x}}_{1},{\hat{x}}_{2}\in H_{D}$), the product of the two is:

(6) $${\hat{x}}_{1}⊗{\hat{x}}_{2}={\tilde{p}}_{1}⊙{\tilde{p}}_{2}+\epsilon \left({\tilde{p}}_{1}⊙{\tilde{q}}_{2}+{\tilde{p}}_{2}⊙{\tilde{q}}_{1}\right).$$

Note that the product is non-commutative.

The dual quaternions apply the same strategy utilized by quaternions, linear operators: ${\left(\cdot \right)}^{+}$ and ${\left(\cdot \right)}^{-}\in R^{8\times 8}$ can be defined for dual quaternions by:

$${\hat{x}}_{1}⊗{\hat{x}}_{2}=Q_{x_{1}}^{+}\cdot {\hat{x}}_{2}=Q_{x_{2}}^{-}\cdot {\hat{x}}_{1},$$

with

$$Q_{x_{1}}^{+}=\left[\begin{array}{cc} Q_{p_{1}}^{+} & O\\ Q_{q_{1}}^{+} & Q_{p_{1}}^{+} \end{array}\right],Q_{x_{2}}^{-}=\left[\begin{array}{cc} Q_{p_{2}}^{-} & O\\ Q_{q_{2}}^{-} & Q_{p_{2}}^{-} \end{array}\right].$$

Dual quaternions have three conjugates [30]: (1) ${\hat{x}}^{1*}=\tilde{p}-\epsilon \tilde{q}$, (2) ${\hat{x}}^{2*}={\tilde{p}}^{*}+\epsilon {\tilde{q}}^{*}$, and (3) ${\hat{x}}^{3*}={\tilde{p}}^{*}-\epsilon {\tilde{q}}^{*}$. A dual quaternion $\hat{x}$ is a unit if $\hat{x}⊗{\hat{x}}^{2*}=1$.

The norm of a dual quaternion in (5) is denoted as $\left|\right|\hat{x}\left|\right|=\sqrt{\hat{x}⊗{\hat{x}}^{2*}}=\sqrt{{\hat{x}}^{2*}⊗\hat{x}}$, which expands to:

$$\begin{array}{c} \left|\right|\hat{x}\left|\right|=\sqrt{\hat{x}⊗{\hat{x}}^{2*}}=\left||p|\right|+\epsilon \frac{\langle p,q\rangle }{\left||p|\right|}. \end{array}$$

The unit dual quaternions have the norm that equals one. A dual quaternion satisfies the unit dual quaternion if and only if the non-dual part ||p|| equals one and the dot product of two parts 〈p,q〉 equals zero. This property helps to understand the relationship between the two parts of the dual quaternion. Especially, a unit quaternion is a unit dual quaternion when the dual part is zero.

### 2.4. Representation of 6D Pose

The 6D pose composes a 3D rotation part represented by a unit quaternion ${\tilde{q}}_{r}=\left[q_{0},q_{1},q_{2},q_{3}\right]$ in the form of (4) and a 3D translation part $t\in R^{3}$. A unit dual quaternion representing the 6D pose is defined by the following equation:

(7) $${\hat{x}}_{d}={\tilde{q}}_{r}+\epsilon {\tilde{q}}_{d},$$

where ${\tilde{q}}_{d}$ for translation t is defined by:

(8) $${\tilde{q}}_{d}=\frac{\tilde{t}⊙{\tilde{q}}_{r}}{2},$$

where $\tilde{t}$ is represented by a purely imaginary translation quaternion with zero real parts.

We introduce the Hamilton operators $H^{+},\;H^{-}$ to replace the linear operators defined above. The term Hamilton operator, which is borrowed from Akyar [31], is not commonly used, at least in the robotics literature, but it seems appropriate here:

$$H_{r}^{-}=\left[\begin{array}{cc} q_{0} & -q^{T}\\ q & -q^{\times }+q_{0}I_{3} \end{array}\right]=\left[\begin{array}{cccc} q_{0} & -q_{1} & -q_{2} & -q_{3}\\ q_{q} & q_{0} & q_{3} & -q_{2}\\ q_{2} & -q_{3} & q_{0} & q_{1}\\ q_{3} & q_{2} & -q_{1} & q_{0} \end{array}\right]$$

so that

(9) $${\tilde{q}}_{d}=\frac{1}{2}H_{t}^{+}{\tilde{q}}_{r}=\frac{1}{2}H_{r}^{-}\tilde{t}.$$

where $H_{t}^{+}$ is a $R^{4\times 4}$ skew-symmetric matrix generated from translation vector t.

A point $p={\left(x,y,z\right)}^{T}\in R^{3}$, we can be embedded in skew-field $H_{D}$ by using a dual quaternion ${\hat{x}}_{d}$ in (7). The transformation can be mathematically described as:

(10) $${\hat{x}}_{d}⊗{\hat{p}}_{d}⊗{\hat{x}}_{d}^{3*}$$

Consistent with the unit quaternion in rotations, the dual quaternions ${\hat{x}}_{d}$ and $-{\hat{x}}_{d}$ represent the same pose.

## 3. Methods

### 3.1. Base Element

Consider the fact that the unit dual quaternions ${\hat{d}}_{q}$ and $-{\hat{d}}_{q}$ in the form of (5) represent the same pose. Furthermore, the underlying structure of the periodic nature of the 6D pose is no longer linear. Hence, a distribution that can characterize the non-linear structure antipodal symmetric property is required. Consequently, we can tackle multiple, conflicting hypotheses that naturally arise in ambiguous situations.

A Bingham distribution [32] is exactly a distribution on a unit hypersphere $S^{d-1}$. The property of hte most interest is the antipodal symmetric, where dF(−x)=dF(x) (i.e., opposite points on $S^{d-1}$ have equal probability). It commonly represents uncertainty on 3D rotations, denoted as SO(3) mathematically, by the unit quaternion form [33], but not on the 6D pose.

**Definition** **1.** *Let a random vector on the hypersphere*$S^{d-1}=\{x\in R^{d}:||x||=1\}\subset R^{d}$*be the unit hypersphere in*$R^{d}$. *The probability density function(p.d.f)*

$$\begin{array}{c} f\;:\;S^{d-1}\to R \end{array}$$

*of a Bingham distribution is given by:*

$$\begin{array}{c} f\left(x\right)=\frac{1}{N}\cdot \exp\left(x^{T}V\Lambda V^{T}x\right) \end{array}$$

*where*$V\in R^{d\times d}$*is an orthogonal matrix* ($VV^{T}=V^{T}V=I_{d\times d}$), *describing the orientation*, $\Lambda =\mathrm{diag}\left(\lambda_{1},\ldots ,\lambda_{d-1},0\right)\in R^{d\times d}$ *with* $\lambda_{1}\leq \cdots \leq \lambda_{d-1}\leq 0$ *as the concentration matrix, and N as a corresponding normalization constant.*

For the representation of small uncertainty, Bingham is almost near the Gaussian distribution, and for the large uncertainty, the Gaussian is worse (or even quite poor) than the Bingham distribution [34].

For those who are familiar with multivariate Gaussian, the parameter matrices V and Λ can be derived via the eigendecomposition of a symmetric matrix C in subsequent Section 3.2 (which is denoted as the inverse covariance matrix $\Sigma^{-1}$ in multivariate Gaussian distribution).

### 3.2. A New Distribution Model

For a quite intuitive interpretation, we decompose a unit dual quaternion $\hat{x}$ into $\left(x_{r},x_{d}\right)$ in vector form. Thus, a joint probability density over the non-dual part, and the dual part can be denoted as the following Lemma 1.

**Lemma** **1.** *A random vector* $x=\left(x_{r},x_{d}\right)\;\in S^{3}\times R^{4}$ *is distributed to the proposed distribution, and the p.d.f is:*

$$\begin{array}{c} f\left(x_{r},x_{d}\right)={N(C)}^{-1}\cdot \exp\left({\left(\begin{array}{c} x_{r}\\ x_{d} \end{array}\right)}^{T}C\left(\begin{array}{c} x_{r}\\ x_{d} \end{array}\right)\right), \end{array}$$

*where* $x_{r}\in S^{3}$ *and* $x_{d}\in R^{4}$, *symmetric and positive definite* $C\in R^{8\times 8}$, *and a normalization constant* N(C).

It is always possible to break a joint density into the product of two factors. We can work out the details for the joint case by employing the *Schur complement*. First, let C in Lemma 1 be denoted as:

$$\begin{array}{c} C=\left(\begin{array}{cc} C_{11} & C_{12}\\ C_{21} & C_{22} \end{array}\right) \end{array}$$

with $C_{ij}\in R^{4\times 4}$; note that $C_{21}=C_{12}^{T}$, according to the settings [22], $C_{11}$ needs to be symmetric, $C_{12}$ may be arbitrary, and $C_{22}$ has to be symmetric negative definite to ensure the antipodal symmetry, which we will further discuss in Section 3.3.

**Lemma** **2.** *The proposed probability density function can be rewritten as:*

$$\begin{array}{cc} f\left(x_{r},x_{d}\right)=N{\left(C\right)}^{-1} & \cdot \exp(x_{r}^{T}A_{1}x_{r}\\ \; & +{\left(x_{d}-A_{2}x_{r}\right)}^{T}C_{22}\left(x_{d}-A_{2}x_{r}\right)) \end{array}$$

*where*$A_{1}=C_{11}-C_{12}C_{22}^{-1}C_{21}$, $A_{2}=-C_{22}^{-1}C_{21}$.

*A proof is given in Appendix A*.

From Lemma 2, the rotation part of the dual quaternion evidently appears as a Bingham distribution, which can be derived by marginalizing out the corresponding conditional distribution of the dual part.

Since the dual part, $x_{d}$, combines the rotation and translation by a Hamilton product, a canonical way to describe dependencies between the position and the orientation of a dual quaternion is still unknown [22].

From (9), the dual part given the non-dual part $f(x_{d}|x_{r})$ can be treated as a multivariate Gaussian distribution $N(A_{2}x_{r},-\frac{C_{22}^{-1}}{2})$. Thus, the joint density function from Lemma 2 can be rewritten as:

$$\begin{array}{cc} f(x_{r},x_{d}) & =f\left(x_{r}\right)f\left(x_{d}|x_{r}\right)\\ \; & ={N_{B}\left(A_{1}\right)}^{-1}\cdot \exp\left(x_{r}^{T}A_{1}x_{r}\right)\cdot \\ \; & \;{N_{G}}^{-1}\cdot \exp\left({\left(x_{d}-A_{2}x_{r}\right)}^{T}C_{22}\left(x_{d}-A_{2}x_{r}\right)\right). \end{array}$$

*where* $A_{1}=C_{11}-C_{12}C_{22}^{-1}C_{21}$, $A_{2}=-C_{22}^{-1}C_{21}$.

### 3.3. Normliazation Constant

The Bingham distribution is flexible for 3D rotation represented by the unit quaternion on the hypersphere $S^{3}$. Although it represents uncertainty, it is still not well propagated in the computer vision and robotics communities because the computation of the normalization constant is complex. The normalization constant, *F*, in the Bingham distribution is not closed form, which means the normalization constant, *F*, only has the numerical solution. However, several techniques can overcome this difficulty, such as caching techniques [35] and saddlepoint approximations. It is still an area of active research [14].

Fortunately, the computational burden is alleviated by the method adapted from [22]. The normalization constant is written as follows according to the discussion:

$$\begin{array}{cc} N(C)= & N_{B}\left(A_{1}\right)\cdot N_{G}=\frac{2\pi \sqrt{\det\left(-\frac{1}{2}C_{22}^{-1}\right)}}{F\left(A_{1}\right)}, \end{array}$$

where $A_{1}=C_{11}-C_{12}C_{22}^{-1}C_{21}$.

Furthermore, this work calculates $N_{B}\left(A_{1}\right)$ by the hypergeometric function [34] of a matrix argument:

$$F\left(\Lambda \right):=\left|S^{d}\right|\cdot {}_{1}F_{1}\left(\frac{1}{2};\frac{d+1}{2};\Lambda \right),$$

For the case d=3, this reduces to:

$$F\left(\Lambda \right)=2\pi^{2}\cdot {}_{1}F_{1}\left(\frac{1}{2};\frac{4}{2};z_{1},z_{2},z_{3}\right).$$

A statistics library [36] is used for the computation of the normalization constant, *F*, in this work.

### 3.4. Parameter Estimation

We hope to obtain the parameters of the proposed distribution given a d×N sample matrix $X=[x_{1},\ldots ,x_{N}]$, where each column vector is assumed to be generated *i.i.d* from our proposed distribution. This procedure can be divided into two stages. First, the first four entries of samples will recover the parameter of the Bingham distribution. Second, the eight entries recover the parameter in the Gaussian distribution.

From Lemma 2, we observed that the base element with d=4 in Section 3.1 appears a marginal distribution of our model. Thus, we can apply Definition 1:

(11) $$f\left(x_{r};\Lambda ,V\right)=\frac{1}{F(\Lambda )}\exp\left({x_{r}}^{T}V\Lambda V^{T}x_{r}\right)$$

where $x_{r}\in R^{4}$ is the first four entries from the proposed distribution.

The parameter V can be obtained as the matrix of eigenvectors of the covariance $A_{1}$ with corresponding eigenvalues Λ in the order $\lambda_{1}\leq \lambda_{2}\leq \lambda_{3}$, equivalent to the eigendecomposition of $A_{1}=V\cdot diag\left(\lambda_{1},\lambda_{2},\lambda_{3}\right)\cdot V^{T}$.

**Figure 2:**
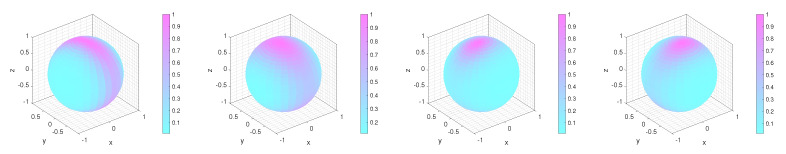
The probability density function of a unit vector under the marginal distribution of the proposed model, which is projected on hypersphere $S^{3}$ with diag(z)=[−4.72,−2.15,−0.60]. The colors encode the value of the probability on the Bingham distribution over the whole sphere.

The probability density function of a 4D Bingham distribution projected on $S^{3}$ by a unit vector is shown in Figure 2, which appears in the marginal distribution of the first four entries $x_{r}$ in our model.

As we have already discussed in Section 3.2, the dual part $x_{d}$ given the non-dual $x_{r}$ is assumed to be a Gaussian distribution $N(A_{2}x_{r},-\frac{C_{22}^{-1}}{2})$. The parameters $A_{2}$ and $-\frac{C_{22}^{-1}}{2}$ can be obtained by maximum likelihood estimation of a multivariate linear regression ([37], Theorem 8.2.1).

The mode of proposed distribution is related to the order of the column vector in matrix V. It turns to be the last column, according to diagonal entries when we enforce $\lambda_{1}\leq \cdots \leq \lambda_{d-1}$ in Λ. It is also possible to swap columns of V without changing the distribution [38]. There exist two correct modes in the proposed distribution because of the antipodal symmetry. For one of the modes $m=(m_{r},m_{d})$, where $m_{r}$ is the normalized eigenvector of $A_{1}$ and $m_{d}=A_{2}m_{r}$, the −m is the another correct one.

## 4. Results

As expected, unit dual quaternions can be used to represent 3D rotation while the dual part is zero. Thus, (4) can be extended to a dual form as:

(12) $${\hat{d}}_{r}=\left[cos\left(\frac{\theta }{2}\right)+sin\left(\frac{\theta }{2}\right)\left(v_{1}i+v_{2}j+v_{3}k\right)\right]+\epsilon \cdot 0$$

where $v=(v_{1},v_{2},v_{3}),||v||=1$.

This work utilizes dual quaternions to generate the form of the 3D translation. A vector $t={\left(t_{1},t_{2},t_{3}\right)}^{T}\in R^{3}$ embedded in the skew-field $H_{D}$ is represented by the following:

(13) $${\hat{d}}_{t}=1+\frac{\epsilon }{2}\left(0+t_{1}i+t_{2}j+t_{3}k\right)$$

To combine rotations and translations, the product of two unit dual quaternions is used according to (6). A rotation with a subsequent translation is given as follows:

(14) $$\begin{array}{cc} {\hat{d}}_{t}⊗{\hat{d}}_{r} & =\left[1+\frac{\epsilon }{2}\left(t_{1}i+t_{2}j+t_{3}k\right)\right]\cdot \left[cos\left(\frac{\theta }{2}\right)+vsin\left(\frac{\theta }{2}\right)\right]\\ \; & =cos\left(\frac{\theta }{2}\right)+sin\left(\frac{\theta }{2}\right)\left(v_{1}i+v_{2}j+v_{3}k\right)+\frac{\epsilon }{2}\{\\ \; & \;-sin\left(\frac{\theta }{2}\right)\left(v_{1}t_{1}+v_{2}t_{2}+v_{3}t_{3}\right)\\ \; & \;+[cos\left(\frac{\theta }{2}\right)t_{1}+sin\left(\frac{\theta }{2}\right)\left(v_{3}t_{2}-v_{2}t_{3}\right)]i\\ \; & \;+[cos\left(\frac{\theta }{2}\right)t_{2}+sin\left(\frac{\theta }{2}\right)\left(v_{1}t_{3}-v_{3}t_{1}\right)]j\\ \; & \;+[cos\left(\frac{\theta }{2}\right)t_{3}+sin\left(\frac{\theta }{2}\right)\left(v_{2}t_{1}-v_{1}t_{2}\right)]k\}\\ \; & =:d_{0}+d_{1}i+d_{2}j+d_{3}k+\epsilon \left(d_{4}+d_{5}i+d_{6}j+d_{7}k\right) \end{array}$$

A dual quaterinon in the form of (14) is still a unit dual quaternion, and there is no restriction on translation that must be unit-length. According to the defined norm property of the unit dual quaternion, that is, $p_{0}q_{0}+p_{1}q_{1}+p_{2}q_{2}+p_{3}q_{3}=0$, this property provides ingredients to our algorithm for translation.

According to (5), the first four entries are interpreted as the non-dual part, which describes the rotation, and its last four entries are interpreted as a dual part of $d_{\hat{x}}$. That is:

(15) $$d_{\hat{x}}={\left(d_{0},d_{1},d_{2},d_{3},d_{4},d_{5},d_{6},d_{7}\right)}^{T}.$$

Represented by the vector form, the unit dual quaternion can be written as $d_{\hat{x}}={\tilde{q}}_{r}+\epsilon {\tilde{q}}_{d}$, decomposing into the non-dual part ${\tilde{q}}_{r}=\left(d_{0},d_{1},d_{2},d_{3}\right)$ and the dual part ${\tilde{q}}_{d}=\left(d_{4},d_{5},d_{6},d_{7}\right)$.

Furthermore, the Hamilton operator ${\left(\cdot \right)}^{+}\in R^{4\times 4}$ is associated with (8), and the translation part can be derived from (14) as follows:

(16) $${\tilde{q}}_{t}=2\cdot {\tilde{q}}_{r}^{*+}\cdot {\tilde{q}}_{d},$$

where ${\tilde{q}}_{r}^{*}=\left(q_{0},-q\right)=\left(d_{0},-d_{1},-d_{2},-d_{3}\right)$ is the conjugate of ${\tilde{q}}_{r}$.

**Figure 3:**
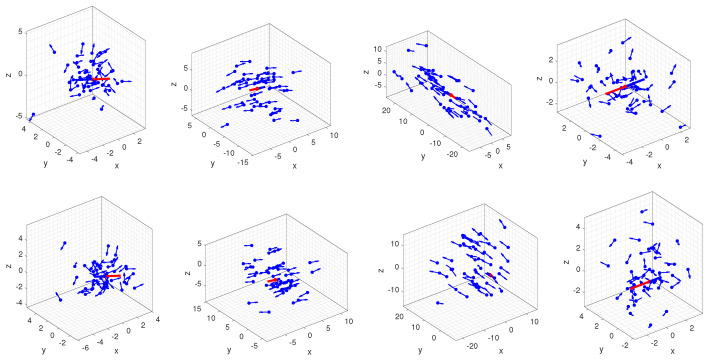
In the first row, the rotation after the translation applies to sets sampled from the proposed distribution, the blue dots mean the position in 3D space, the rotation axis is represented by an arrow. In the bottom row, set the translation after the rotation. The mode in both rows appears as the antipodal symmetry in the representation of rotation; there is no such property in the position that recovers from the proposed distribution (Zoom in the figure to see the detail).

Algorithm  1 shows a robust method to generate the rotation angle, axis, and translation in 3D space by sampling from the proposed distribution. Furthermore, this work applies Algorithm 1 to several sets of the samples generated from the proposed distribution, and the results are shown in Figure 3.
**Algorithm 1:** Recover the corresponding translation and rotation from sampled vector on the proposed distribution.**Input**: A sampled random vector $w_{\hat{x}}={\left(w_{0},\ldots ,w_{7}\right)}^{T}\in R^{8}$
/* 
(Compute the rotation angle(
*/$\phi \leftarrow 2\cdot atan2(\sqrt{{w_{1}}^{2}+{w_{2}}^{2}+{w_{3}}^{2}},w_{0})$
/* 
(Compute the rotational arbitrary vector(
*/$v_{1}\leftarrow w_{1}/sin\left(\phi /2\right)$
$v_{2}\leftarrow w_{2}/sin\left(\phi /2\right)$
$v_{3}\leftarrow w_{3}/sin\left(\phi /2\right)$
/* (Compute the translation(
*/$t_{1}\leftarrow 2\cdot (w_{0}w_{5}-w_{1}w_{4}+w_{2}w_{7}-w_{3}w_{6})$
$t_{2}\leftarrow 2\cdot (w_{0}w_{6}-w_{1}w_{7}-w_{2}w_{4}+w_{3}w_{5})$
$t_{3}\leftarrow 2\cdot (w_{0}w_{7}+w_{1}w_{6}-w_{2}w_{5}-w_{3}w_{4})$
**Output**: Rotation angle ϕ, rotational arbitrary vector $v=(v_{1},v_{2},v_{3})$ and translation vector $t=(t_{1},t_{2},t_{3})$


The 6D pose is obtained by the random vector sampled from the proposed distribution. Each sample from our distribution appears in the antipodal symmetry in rotations. Compared with the planar rigid motion case [22], our distribution breaks the antipodal symmetry in the translation that t and −t are denoted as positions that always have the same probability values.

## 5. Conclusions

This work proposes a distribution over the 6D pose in order to capture the uncertainty which is typically ignored by the pose estimation task. Our model takes the periodic nature of the underline structure into consideration, such as antipodal symmetry, which is suitable for high-noise regimes. The proposed distribution eliminates the drawbacks from initializing the SE(2), in which the position is ambiguous due to antipodal symmetry, and our distribution setting is closer to reality.

However, we assume that the Gaussian on the correlation part leads to a better, but still imperfect, answer. Many beautiful properties in the parameter matrix of the proposed distribution have not been explored. Still, a unified distribution exists to couple the rotation and translation.

In the future, we will verify the proposed distribution in practical applications, such point cloud registration, as well as the most important one, the 6D pose estimation task. Last but not least, the combination of the neural network with the proposed probability to regress the object pose is worth endeavoring.

## Data Availability

Data sharing is not applicable to this article.

## Appendix A. Proof of Lemma 2

The parameter matrix ***C*** in the distribution can be rewritten as the UDL form by *Schur Complement*:

$$\begin{array}{cc} {\left(\begin{array}{c} x_{r}\\ x_{d} \end{array}\right)}^{T} & \left(\begin{array}{cc} C_{11} & C_{12}\\ C_{21} & C_{22} \end{array}\right)\left(\begin{array}{c} x_{r}\\ x_{d} \end{array}\right)\\ ={\left(\begin{array}{c} C_{r}\\ x_{d} \end{array}\right)}^{T} & \left(\begin{array}{cc} 1 & C_{12}C_{22}^{-1}\\ 0 & 1 \end{array}\right)\left(\begin{array}{cc} C_{11}-C_{12}C_{22}^{-1}C_{21} & 0\\ 0 & C_{22} \end{array}\right)\left(\begin{array}{cc} 1 & 0\\ C_{22}^{-1}C_{21} \end{array}\right)\left(\begin{array}{c} x_{r}\\ x_{d} \end{array}\right)\\ & =x_{r}^{T}\left(C_{11}-C_{12}C_{22}^{-1}C_{21}\right)x_{r}+{\left(x_{d}+{C_{22}}^{-1}C_{21}x_{r}\right)}^{T}C_{22}\left(x_{d}+{C_{22}}^{-1}C_{21}x_{r}\right) \end{array}$$

where, once again, $A_{1}=C_{11}-C_{12}C_{22}^{-1}C_{21}$, $A_{2}=-C_{22}^{-1}C_{21}$.

## References

[1] Lin H.Y., Liang S.C., Chen Y.K. (2021). Robotic Grasping with Multi-View Image Acquisition and Model-Based Pose Estimation. IEEE Sens. J..

[2] Zhu Q., Xie H., Cai C., Liu P. A rapid and precise self-localization approach of mobile robot based on binocular omni-directional vision. Proceedings of the 2017 36th Chinese Control Conference (CCC).

[3] Barfoot T.D. (2017). State Estimation for Robotics.

[4] Zeng A., Song S., Yu K.T., Donlon E., Hogan F.R., Bauza M., Ma D., Taylor O., Liu M., Romo E. Robotic Pick-and-Place of Novel Objects in Clutter with Multi-Affordance Grasping and Cross-Domain Image Matching. Proceedings of the 2018 IEEE International Conference on Robotics and Automation (ICRA).

[5] Krizhevsky A., Sutskever I., Hinton G.E. (2012). Imagenet classification with deep convolutional neural networks. Adv. Neural Inf. Process. Syst..

[6] Deng J., Dong W., Socher R., Li L.J., Li K., Li F.-F. Imagenet: A large-scale hierarchical image database. Proceedings of the 2009 IEEE Conference on Computer Vision and Pattern Recognition (CVPR).

[7] Shamsfakhr F., Bigham B.S. (2020). GSR: Geometrical scan registration algorithm for robust and fast robot pose estimation. Assem. Autom..

[8] Okorn B., Xu M., Hebert M., Held D. Learning Orientation Distributions for Object Pose Estimation. Proceedings of the 2020 IEEE/RSJ International Conference on Intelligent Robots and Systems (IROS).

[9] Xiang Y., Schmidt T., Narayanan V., Fox D. PoseCNN: A Convolutional Neural Network for 6D Object Pose Estimation in Cluttered Scenes. Proceedings of the Robotics: Science and Systems (RSS).

[10] Wang C., Xu D., Zhu Y., Martín-Martín R., Lu C., Li F.-F., Savarese S. DenseFusion: 6D Object Pose Estimation by Iterative Dense Fusion. Proceedings of the 2019 IEEE/CVF Conference on Computer Vision and Pattern Recognition (CVPR).

[11] Hodaň T., Baráth D., Matas J. EPOS: Estimating 6D Pose of Objects With Symmetries. Proceedings of the 2020 IEEE/CVF Conference on Computer Vision and Pattern Recognition (CVPR).

[12] Manhardt F., Arroyo D.M., Rupprecht C., Busam B., Birdal T., Navab N., Tombari F. Explaining the Ambiguity of Object Detection and 6D Pose From Visual Data. Proceedings of the 2019 IEEE/CVF International Conference on Computer Vision (ICCV).

[13] Deng X., Mousavian A., Xiang Y., Xia F., Bretl T., Fox D. PoseRBPF: A Rao-Blackwellized Particle Filter for 6D Object Pose Tracking. Proceedings of the Robotics: Science and Systems.

[14] Gilitschenski I., Sahoo R., Schwarting W., Amini A., Karaman S., Rus D. Deep Orientation Uncertainty Learning based on a Bingham Loss. Proceedings of the International Conference on Learning Representations (ICLR).

[15] Prokudin S., Gehler P., Nowozin S. Deep Directional Statistics: Pose Estimation with Uncertainty Quantification. Proceedings of the 2018 European Conference on Computer Vision (ECCV).

[16] Daniilidis K. (1999). Hand-Eye Calibration Using Dual Quaternions. Int. J. Robot. Res..

[17] Goddard J.S., Abidi M.A., Ellson R.N., Nurre J.H. (1998). Pose and Motion Estimation Using Dual Quaternion-Based Extended Kalman Filtering. Three-Dimensional Image Capture and Applications.

[18] Horn B., Hilden H., Negahdaripour S. (1988). Closed-Form Solution of Absolute Orientation using Orthonormal Matrices. J. Opt. Soc. Am. A.

[19] Srivatsan R.A., Xu M., Zevallos N., Choset H. (2018). Probabilistic pose estimation using a Bingham distribution-based linear filter. Int. J. Robot. Res..

[20] Li K., Pfaff F., Hanebeck U.D. Geometry-Driven Stochastic Modeling of SE(3) States Based on Dual Quaternion Representation. Proceedings of the 2019 IEEE International Conference on Industrial Cyber Physical Systems (ICPS).

[21] Glover J., Popovic S. Bingham Procrustean Alignment for Object Detection in Clutter. Proceedings of the 2013 IEEE/RSJ International Conference on Intelligent Robots and Systems.

[22] Gilitschenski I., Kurz G., Julier S.J., Hanebeck U.D. A New Probability Distribution for Simultaneous Representation of Uncertain Position and Orientation. Proceedings of the 17th International Conference on Information Fusion (FUSION).

[23] Corke P. (2017). Robotics, Vision and Control.

[24] Jackson B.E., Tracy K., Manchester Z. (2021). Planning with Attitude. IEEE Robot. Autom. Lett..

[25] Feiten W., Atwal P., Eidenberger R., Grundmann T. (2009). 6D Pose Uncertainty in Robotic Perception.

[26] Stuelpnagel J. (1964). On the Parametrization of the Three-Dimensional Rotation Group. SIAM Rev..

[27] Feiten W., Lang M., Hirche S. Rigid Motion Estimation Using Mixtures of Projected Gaussians. Proceedings of the 16th International Conference on Information Fusion (FUSION).

[28] Kenwright B. A Beginners Guide to Dual-Quaternions: What They Are, How They Work, and How to Use Them for 3D Character Hierarchies. Proceedings of the 20th International Conference on Computer Graphics, Visualization and Computer Vision, WSCG 2012 Communication Proceedings.

[29] Kavan L., Collins S., Žára J., O’Sullivan C. (2007). Skinning with Dual Quaternions. I3D ’07: Proceedings of the 2007 Symposium on Interactive 3D Graphics and Games.

[30] Fan T., Weng H., Murphey T. Decentralized and Recursive Identification for Cooperative Manipulation of Unknown Rigid Body with Local Measurements. Proceedings of the 2017 IEEE 56th Annual Conference on Decision and Control (CDC).

[31] Akyar B. (2008). Dual Quaternions in Spatial Kinematics in an Algebraic Sense. Turk. J. Math..

[32] Bingham C. (1974). An Antipodally Symmetric Distribution on the Sphere. Ann. Stat..

[33] Antone M.E. (2001). Robust Camera Pose Recovery Using Stochastic Geometry. Ph.D. Thesis.

[34] Kurz G., Gilitschenski I., Julier S., Hanebeck U.D. Recursive Estimation of Orientation Based on the Bingham Distribution. Proceedings of the 16th International Conference on Information Fusion (FUSION).

[35] Glover J., Bradski G., Rusu R.B., Park M. (2012). Monte Carlo Pose Estimation with Quaternion Kernels and the Bingham Distribution. Robot. Sci. Syst..

[36] Kurz G., Gilitschenski I., Pfaff F., Drude L., Hanebeck U.D., Haeb-Umbach R., Siegwart R.Y. (2019). Directional Statistics and Filtering Using libDirectional. J. Stat. Softw. Artic..

[37] Anderson T.W. (2003). An Introduction to Multivariate Statistical Analysis.

[38] Kurz G., Gilitschenski I., Julier S., Hanebeck U. (2014). Recursive Bingham Filter for Directional Estimation Involving 180 Degree Symmetry. J. Adv. Inf. Fusion.

